# Functional Differences between Mitochondrial Haplogroup T and Haplogroup H in HEK293 Cybrid Cells

**DOI:** 10.1371/journal.pone.0052367

**Published:** 2012-12-26

**Authors:** Edith E. Mueller, Susanne M. Brunner, Johannes A. Mayr, Olaf Stanger, Wolfgang Sperl, Barbara Kofler

**Affiliations:** 1 Research Program for Receptor Biochemistry and Tumor Metabolism, Department of Pediatrics, Paracelsus Medical University, Salzburg, Austria; 2 Department of Cardiac Surgery, Paracelsus Medical University, Salzburg, Austria; Ben-Gurion University of the Negev, Israel

## Abstract

**Background:**

Epidemiological case-control studies have revealed associations between mitochondrial haplogroups and the onset and/or progression of various multifactorial diseases. For instance, mitochondrial haplogroup T was previously shown to be associated with vascular diseases, including coronary artery disease and diabetic retinopathy. In contrast, haplogroup H, the most frequent haplogroup in Europe, is often found to be more prevalent in healthy control subjects than in patient study groups. However, justifications for the assumption that haplogroups are functionally distinct are rare. Therefore, we attempted to compare differences in mitochondrial function between haplogroup H and T cybrids.

**Methodology/Principal Findings:**

Mitochondrial haplogroup H and T cybrids were generated by fusion of HEK293 cells devoid of mitochondrial DNA with isolated thrombocytes of individuals with the respective haplogroups. These cybrid cells were analyzed for oxidative phosphorylation (OXPHOS) enzyme activities, mitochondrial DNA (mtDNA) copy number, growth rate and susceptibility to reactive oxygen species (ROS). We observed that haplogroup T cybrids have higher survival rate when challenged with hydrogen peroxide, indicating a higher capability to cope with oxidative stress.

**Conclusions/Significance:**

The results of this study show that functional differences exist between HEK293 cybrid cells which differ in mitochondrial genomic background.

## Introduction

Mitochondria provide most of the energy in a cell by a process called oxidative phosphorylation (OXPHOS), where carbohydrates and fats are oxidized by oxygen to produce carbon dioxide, water and adenosine triphosphate (ATP) [1], [2].

Although the majority of mitochondrial proteins are encoded by nuclear DNA and imported into the mitochondria, these multi-functional organelles also contain their own DNA (mitochondrial DNA, mtDNA). Twenty-four genes of the mtDNA code for components of the mitochondrial translational machinery (2 ribosomal RNAs and 22 transfer RNAs) and 13 genes provide essential subunits of the energy-generating enzymes of the OXPHOS pathway, namely complex I, III, IV and V. Only succinate dehydrogenase (complex II) is completely composed of nuclear encoded subunits [1]–[3].

Mitochondrial function declines with age, and both mtDNA alterations and oxidative damage accumulate. Oxidative damage is produced by reactive oxygen species (ROS), and most of the cellular ROS, such as superoxide, hydrogen peroxide and organic hydroperoxides, are generated in mitochondria from single electrons escaping the mitochondrial respiratory chain and reducing molecular oxygen [2]. An electron leak is produced either when OXPHOS is inhibited and electron transfer is defective or when an overload of nutritional intake in combination with tightly coupled mitochondria causes electrons to accumulate [4].

During evolution, the human population accumulated a high number of mtDNA base substitutions along radiating maternal lineages, where specific combinations of polymorphisms constitute what are referred to as mitochondrial haplogroups. Researchers have used and continue to use these population-specific polymorphisms to elucidate long-ago human migrations and human pre-history [2]. It is believed that mtDNA variants and mitochondrial haplogroups differ in their OXPHOS performance, energy consumption and heat production, differences which may have allowed humans to adapt to climatic and nutritional changes [5].

However, mitochondrial haplogroups have also been shown to be associated with multifactorial diseases [6]–[11]. In our laboratory, mitochondrial haplogroup T was found to be associated with coronary artery disease (CAD) and diabetic retinopathy [12]. Haplogroup Twas also found to correlate with reduced spermatozoa motility [8], hypertrophic cardiomyopathy [9], age-related macular degeneration (AMD) [13], [14] and type 2 diabetes mellitus [15], and to be negatively associated with the status of elite endurance athletes [16]. A study by Baudouin et al. revealed that individuals with haplogroup H have a two-fold increased chance of survival after sepsis compared to individuals with other haplogroups [17]. Haplogroup H was also discovered to be significantly more abundant in individuals with normal sperm motility [8], in healthy subjects compared to patients with CAD [12], and in healthy subjects compared to patients with choroidal neovascularization (CNV) [11], and to be associated with a reduced prevalence of age-related maculopathy [18]. However, there are also studies showing that mitochondrial haplogroup H is associated with illness and haplogroup T is associated with protection. For instance, in epidemiological studies on Alzheimer’s disease (AD), haplogroup T was found to be underrepresented in AD [19], whereas haplogroup H or its subhaplogroup H5 were detected to be risk factors [20]–[22].

Mitochondrial polymorphisms defining mitochondrial haplogroups or definite combinations of such polymorphisms could slightly alter OXPHOS coupling and performance as well as ROS production.

To exclude the influence of a nucleus–mtDNA genetic interaction, an excellent in vitro system to study functional differences of mtDNA variations is the use of cybrids. Transmitochondrial cybrids are produced by fusion of cells depleted of their mtDNA (ρ^0^ cells) with cells devoid of nuclear DNA (e.g. thrombocytes). This technique is very useful to discriminate the functional consequences of mtDNA variations from the nuclear background [23]. Cybrids have already been successfully used to distinguish the consequences of mtDNA mutations [24], [25] and polymorphisms [26], and also to find dissimilarity between mitochondrial haplogroups [27]–[30].

Our aim was to elucidate functional differences between the European mitochondrial haplogroups H and T. The activity of mitochondrial OXPHOS enzymes, mtDNA copy number, proliferation capacity as well as susceptibility to oxidative stress were compared between haplogroup H- and T-specific cybrids.

## Results

Based on our previous observation that patients with mitochondrial haplogroup T have a higher risk of developing CAD [12], and because haplogroup H is the most frequent haplogroup in Europe, we decided to compare cybrid cells of haplogroups H and T in terms of their mitochondrial functions. Cybrids were produced by fusion of HEK293 ρ^0^ cells with thrombocytes of three healthy individuals with mitochondrial haplogroup H (HEK H cybrids) and with thrombocytes of three patients with CAD with mitochondrial haplogroup T (HEK T cybrids) [31].

### MtDNA Sequence Variation of Cybrids

**Figure 1:**
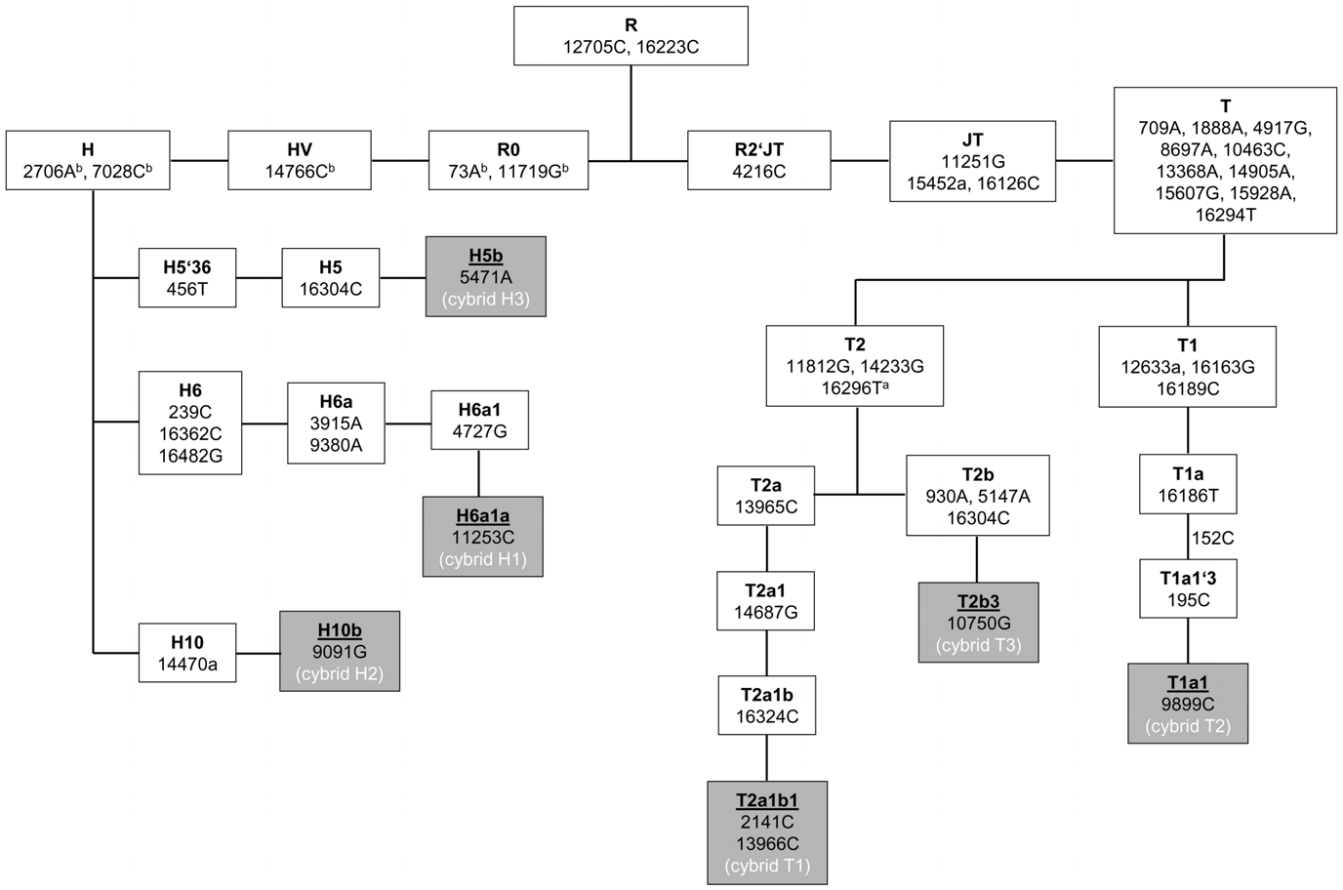
Figure 1. Phylogenetic tree of haplogroup H and T subsets. The phylogenetic tree was constructed according to phylotree.org [32]. ^a^C16296T did not appear in the mtDNA sequence of cybrid T1. ^b^Bases of the Revised Cambridge Reference Sequence that appear in HEK T cybrids as polymorphisms diagnostic for non-H haplogroups (m.73A>G, m.2706A>G, m.7028C>T, m.11719G>A and m.14766C>T).

Whole mtDNA sequencing of cybrids was performed and individual polymorphisms (compared to the Cambridge Reference Sequence) were used to construct a phylogenetic tree of sub-haplogroups, according to phylotree.org [32]. HEK H cybrids were classified into the sub-haplogroups H5b, H6a1a and H10b and HEK T cybrids were classified into the sub-haplogroups T1a1, T2a1b1 and T2b3 (Figure 1). Comparison of the mtDNA sequences to the Cambridge Reference Sequence revealed eight non-haplogroup-defining polymorphisms, which were present in all cybrids (m.263A>G; m.309-m.310insC or m.309-m.310insCC; m.315insC; m.750A>G; m.1438A>G; m.4769A>G; m.8860A>G; m.15326A>G).

Non-haplogroup-defining polymorphisms not present in all cybrids are listed in Supplementary Table S1. Most differences between haplogroup specific cybrids concern noncoding regions of the mtDNA. Seven polymorphisms were detected in D-Loop Regions. m.16519T>C and m.152T>C are very common polymorphisms, with frequencies of 59.7% and 21.2% in the population (www.genpat.uu.se/mtDB/, [33]) and found in four out of six and three out of six cybrids, respectively. The D-Loop polymorphisms m.151C>T, m.16184C>T, m.16344C>T are also common, at a frequency of at least one percent in the population (www.genpat.uu.se/mtDB/, [33]). Only two of the D-Loop polymorphism (m.573-m.574insC, m.16280A>G) have not been described in the Human Mitochondrial Genome Database [33]. The insertion of cysteins in a poly-C stretch between position 568 and 573 (m.573-m.574insC, cybrid T2) is described to occur in 27 sub-haplogroups [32] and position m.16280A>G (cybrid T3) has nine citations [34]–[42] in Mitomap (www.mitomap.org, [43]), without reported association to disease. The gain of one cystein at 5895–5899, in the short non-coding region between the tRNA tyrosine and MT-CO1 genes, detected in cybrid H1, was described in one subject with progressive external ophthalmoplegia [44]. Four sequence variations were found in mitochondrial ribosomal RNA genes. In the 16s rRNA, the heteroplasmic position m.2170G>A with 48% substitution rate, as well as the base substitutions m.2412A>G and m.1760G>A, have not been reported previously [33], [43]. Position m.1598G>A in the 12 s rRNA, found in cybrid T2, is occurring with a frequency of 2.5% in the Human Mitochondrial Genome Database [33]. Position m.12696T>C (0.3%, [33]) detected in cybrid T1, affects complex I subunit ND5, but is a synonymous mutation. It is described to be a haplogroup-defining polymorphism in sub-haplogroups L0d1a1, M33a1b, HV1b, H56b and U5b2b1a2 [32]. Only two polymorphisms in cybrids H2 and one in T2 lead to differences in the amino acid compositions. The amino-acid change at position m.14324T>C in the MT-ND6 gene found in cybrid T2 has already been described, however not as a disease associated mutation but to occur in haplogroup C1e [45]. The relevant amino acid p.Asn117 is not conserved across vertebrates. The two heteroplasmic variants found in cybrid H2, m.6996A>C (approximately 50% heteroplasmy) in the MT-CO1 gene and m.15246G>A (approximately 25% heteroplasmy) affect conserved amino acids in the MT-CYB gene and have not been reported previously [33], [43].

### Normalized Activities of OXPHOS Enzymes do not Differ between H and T

Because mtDNA encodes subunits of the OXPHOS system, variations of mtDNA could primarily affect the activity of OXPHOS enzymes. We determined possible functional differences between mitochondrial haplogroup H and T in HEK293 cybrids by measuring the enzymatic activities of OXPHOS enzymes and the tricarboxylic acid (TCA) cycle enzyme citrate syntase (CS) as a reference [31].

There were no significant differences between HEK H and T cybrids in the enzymatic activities of CS or of OXPHOS complexes (Table 1).

**Table 1:** Enzymatic activities of citrate synthase and oxidative phosphorylation complexes I – V in haplogroup H and T cybrid cells.

	Haplogroup H^a^	Haplogroup T^a^	P-value^b^
	n = 3	n = 3	
Citrate synthase (mUnits/mg protein)^c^	1089.2 (228.4)	1005.8 (236.6)	0.683
Complex I (mUnits/mg protein)	17.7 (1.4)	18.7 (2.4)	0.562
Complex I (mUnits/mUnits CS)	0.017 (0.004)	0.020 (0.007)	0.492
Complex I+III (mUnits/mg protein)	92.0 (23.4)	131.4 (26.5)	0.125
Complex I+III (mUnits/mUnits CS)	0.088 (0.033)	0.133 (0.008)	0.076
Complex II (mUnits/mg protein)^c^	243.7 (34.3)	215.8 (43.9)	0.434
Complex II (mUnits/mUnits CS)	0.232 (0.037)	0.244 (0.051)	0.750
Complex II+III (mUnits/mg protein)	431.8 (84.7)	356.2 (105.3)	0.388
Complex II+III (mUnits/mUnits CS)	0.407 (0.010)	0.393 (0.120)	0.889
Complex III (mUnits/mg protein)	451.9 (98.5)	420.1 (169.5)	0.792
Complex III (mUnits/mUnits CS)	0.408 (0.095)	0.454 (0.275)	0.795
Complex IV (mUnits/mg protein)	412.1 (55.1)	316.5 (96.3)	0.210
Complex IV (mUnits/mUnits CS)	0.376 (0.085)	0.349 (0.157)	0.809
Complex V (mUnits/mg protein)	195.1 (46.6)	132.1 (33.6)	0.130
Complex V (mUnits/mUnits CS)	0.183 (0.021)	0.149 (0.037)	0.245

a Values are given as mean ± standard deviation (SD).

b P-value: Independent samples t-test.

c Reported previously in Figure 2 of [31].

Enzymatic activity measurements were made on isolated mitochondria of cells grown in glucose medium with antibiotics, and on cells with five to 15 passages after the cybridization process.

### MtDNA Copy Number is Higher in Haplogroup T Cybrids

Because mtDNA copy number is a reliable indicator of OXPHOS activity, we further analyzed mtDNA copy number in haplogroup-specific cybrid cells. Due to the fact that the inter-assay variation associated with the determination of mtDNA copy number is lower than the variation associated with the determination of OXPHOS enzyme activities, we hypothezised that true variation in mtDNA copy number has a better chance to impart subtle but significant differences among haplogroup-specific cybrids.

For determination of mtDNA copy number, the cells were cultivated in glucose or galactose medium. High concentrations of glucose in the cultivation medium frequently leads to inhibition of respiration (crabtree effect) [46]. Cultivation of cells in non-glucose containing galactose medium hence might increase respiration, and haplogroup dependent OXPHOS differences may be more prominent.

**Figure 2:**
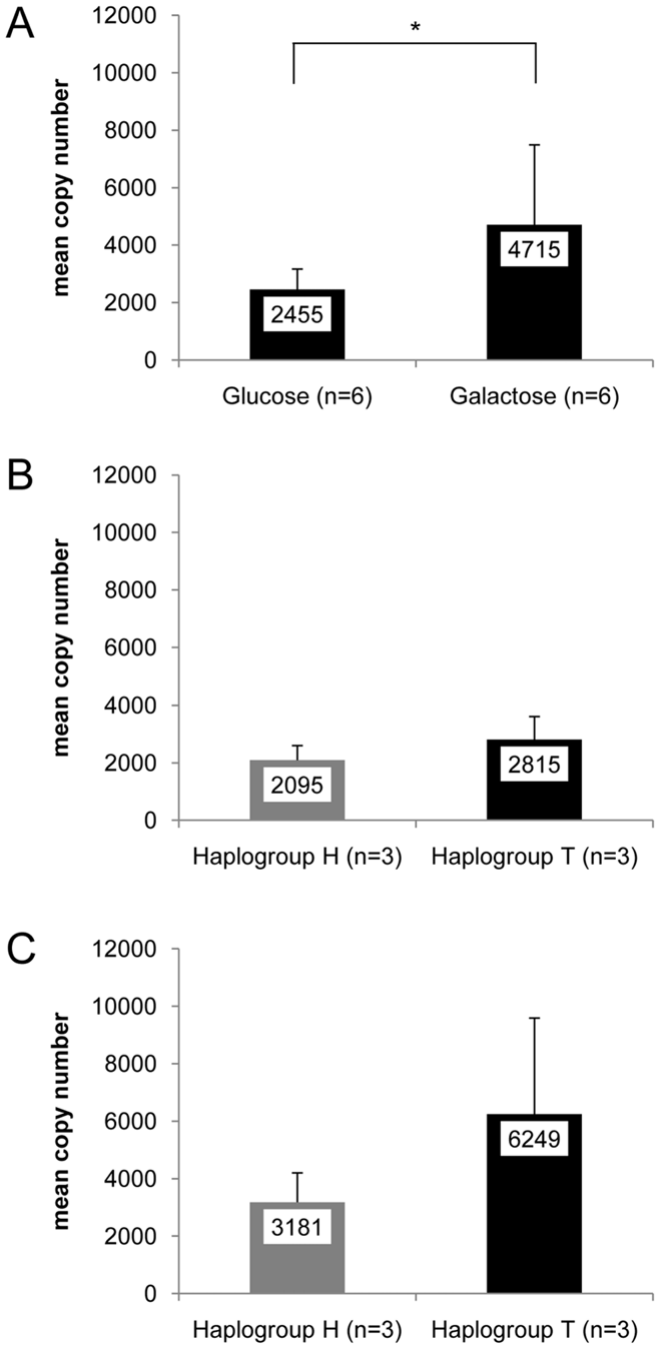
Mitochondrial DNA copy number in cybrid cells. (A) Comparison of all cybrid clones cultivated in glucose medium and galactose medium. (B) Comparison of HEK H and HEK T cybrids cultivated in glucose medium. (C) Comparison of HEK H and HEK T cybrids cultivated in galactose medium. Mean values of copy numbers are given; error bars: standard deviation; *p<0.05.

As expected, cultivation of cells in galactose medium resulted in a significantly higher mtDNA copy number compared to cells cultivated in medium supplemented with glucose (Figure 2A). We detected a higher mtDNA copy number in HEK T cybrids compared to HEK H cybrids, with the difference being more pronounced in cybrids cultivated in galactose medium (Figures 2B and C). However, these differences were statistically not significant.

### Haplogroup T Cybrids have a Higher Growth Rate

The proliferation capacity of cells mirrors their energy-producing capacity. Hence, we next investigated the proliferation rate of HEK H and T cybrid cells by two different methods.

#### Growth curves

Growth curves were determined by measuring the number of HEK H and HEK T cybrid cells using a CyQUANT® NF Cell Proliferation Assay Kit.

**Figure 3:**
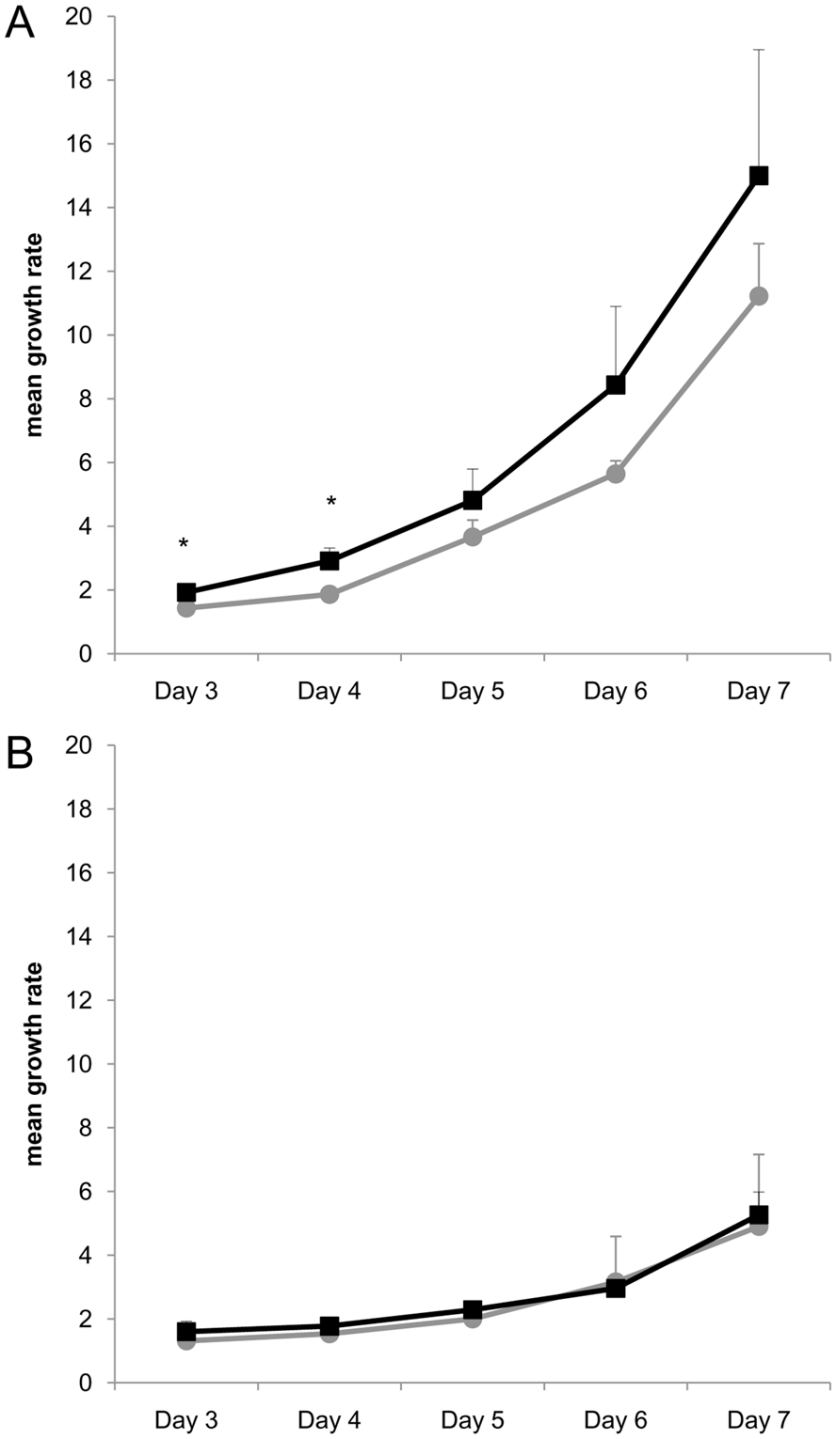
Growth curves of mitochondrial haplogroup-specific cybrid cells. The number of cells on the days given were normalized to the number of cells on day two and determined as growth rate. (A) Comparison of HEK H (n = 3; gray circles) and HEK T (n = 3; black squares) cybrids at days three to seven in glucose medium. (B) Comparison of HEK H (n = 3; gray circles) and HEK T (n = 3; black squares) cybrids at days three to seven in galactose medium. Mean values of growth rates are given; error bars: standard deviation; *p<0.05.

The mean growth rate of the cybrid cells was higher on days three to seven for HEK T compared to HEK H cybrids when cultivated in glucose medium, and significant p-values were obtained on days three and four (Figure 3A). In galactose medium, the growth of HEK H and HEK T cybrids did not differ significantly (Figure 3B).

#### Competitive mix experiments

A more sensitive method of comparing the growth capability of different cells is a competitive mix experiment. The prolonged cultivation time and direct competition of cybrids within the same culture flask allows a direct comparison of growth rates. Each HEK H cybrid clone was co-cultivated with each HEK T cybrid clone at a 1∶1 mixture of cells. After 10, 20 and 30 days of co-culture, DNA was isolated and the proportion of each genotype was analyzed using TaqMan quantitative real-time PCR (qPCR) with probes specific for haplogroup H (7028C) or all other haplogroups (7028T, in our case indicative for haplogroup T).

**Figure 4:**
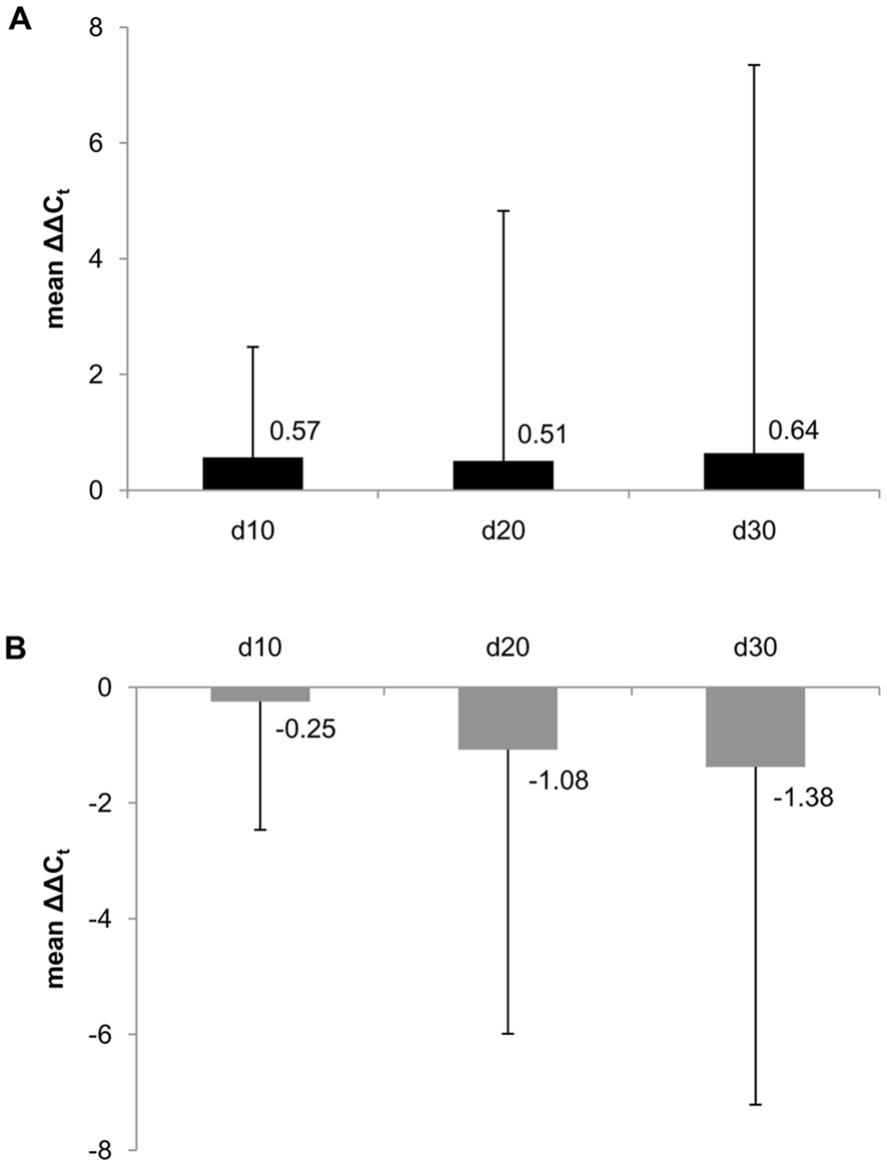
Results of TaqMan qPCR analysis of HEK H and HEK T cybrid competitive co-cultures. After 10, 20 and 30 days (d10, d20, d30) of co-culture, isolated DNA of the cell mixtures was analyzed using TaqMan qPCR. ΔC_t_ values were calculated by subtraction of the mean C_t_ value of the FAM signal (probe recognizing haplogroup T) from the mean C_t_ value of the VIC signal (probe recognizing haplogroup H). ΔΔC_t_ values were calculated by subtraction of the mean ΔC_t_ values of the original cell mixtures (n = 36; day zero) from the mean ΔC_t_ values of all co-cultures at days 10, 20 or 30 (n = 36; except for d30 in galactose: n = 35). Dominance of haplogroup H results in a negative ΔΔC_t_ value and is presented as gray bars, whereas dominance of haplogroup T results in a positive ΔΔC_t_ value and is presented as black bars. (A) ΔΔC_t_ values of competitive co-cultures cultivated in glucose medium, at days 10, 20 and 30. (B) ΔΔC_t_ values of competitive co-cultures cultivated in galactose medium, at days 10, 20 and 30. Mean ΔΔC_t_ values are given; error bars: standard deviation.

After 10, 20 and 30 days in glucose medium, a trend toward haplogroup T as the dominant genotype was observed (Figure 4A), whereas in galactose medium a trend toward dominance of haplogroup H was observed (Figure 4B).

### Mitochondrial Haplogroup T is Less Susceptible to Oxidative Stress

Subtle differences between haplogroup-specific cybrids might only become apparent under stress conditions. Therefore, we challenged the cybrids with different concentrations of hydrogen peroxide (H_2_O_2_) and measured susceptibility to ROS by determination of cell viability.

**Figure 5:**
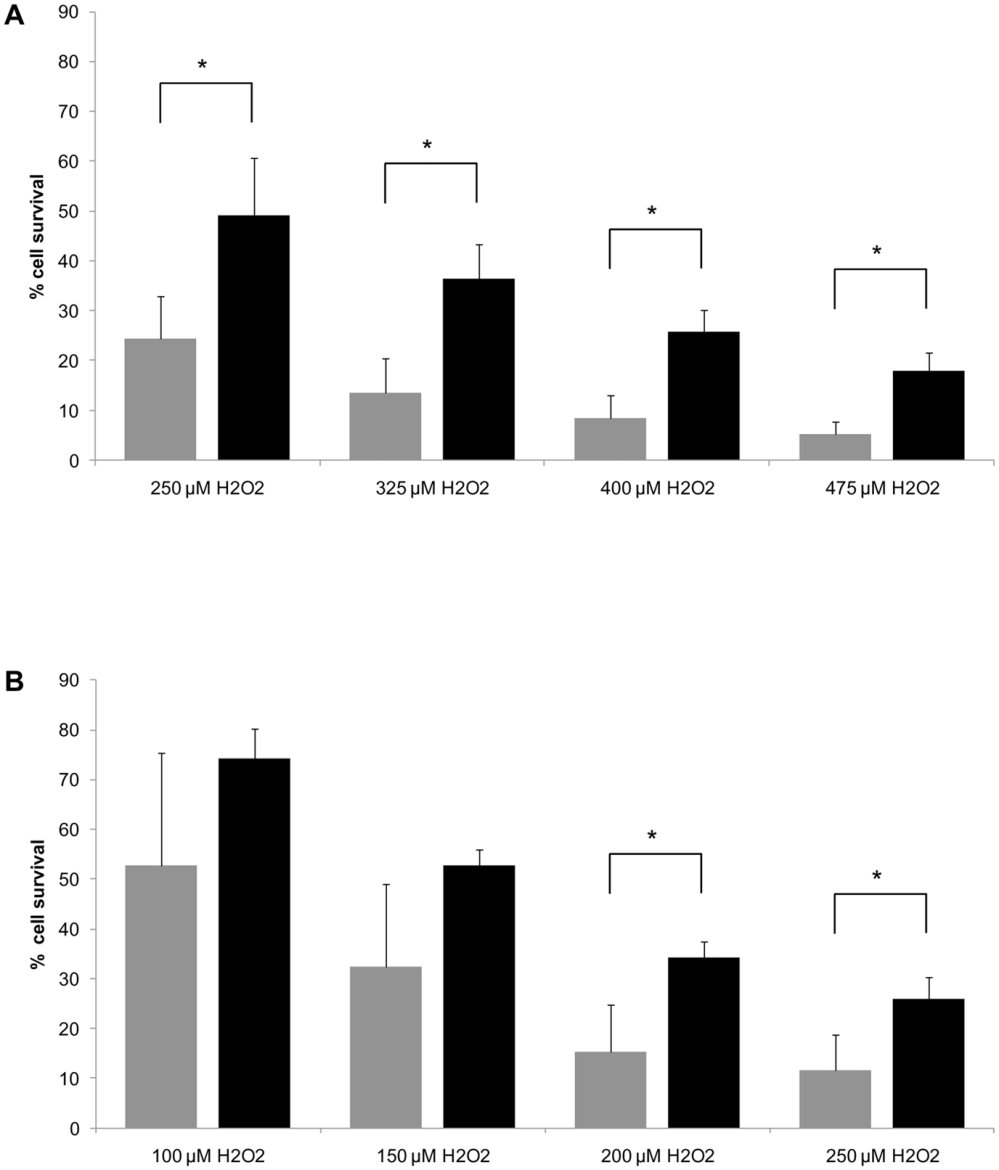
Cell survival after treatment with H_2_O_2_. Cell survival was measured 24 hours after H_2_O_2_ treatment and calculated as a percentage of the ratio between treated and untreated cells (% cell survival). (A) Comparison of HEK H (n = 3; gray bars) and HEK T (n = 3; black bars) cybrids at 250 µM to 475 µM H_2_O_2_ in glucose medium without serum and without sodium pyruvate. (B) Comparison of HEK H (n = 3; gray bars) and HEK T (n = 3; black bars) cybrids at 100 µM to 250 µM H_2_O_2_ in galactose medium without serum and without sodium pyruvate. Mean values of % cell survival are given; error bars: standard deviation; *p<0.05.

Cells grown in galactose medium were more sensitive to H_2_O_2_ treatment than cells grown in glucose medium (Figure 5).

Cell survival of HEK T cybrids was higher than that of HEK H cybrids. Statistically significant differences were found at all four concentrations of H_2_O_2_ in glucose medium as well as in the two highest concentrations (200 µM, 250 µM) of H_2_O_2_ in galactose medium (Figure 5A and B).

## Discussion

Previous studies showing mitochondrial haplogroup T to be a risk factor for CAD and diabetic retinopathy, as well as other literature, motivated us to elucidate functional differences between haplogroups H and T. Haplogroup T was found to be a risk factor for developing peripheral neuropathy during antiretroviral therapy [10] and AMD [13], [14] and to be protective for AD [19]. Haplogroup H, however, was found to be a protective factor for AMD [11], [18] and for outcome in sepsis [17], but to be significantly associated with the risk of developing AD [20]–[22]. Therefore, we aimed to generate transmitochondrial cybrid cell lines and hypothesized that cybrids derived from patients with haplogroup T differ from cybrids derived from healthy controls with haplogroup H in their properties related to mitochondrial functions.

Some attempts have already been made to determine differences between haplogroup H and other haplogroups in cybrid cells. Carelli et al. compared the European haplogroups H, T and J in cybrids derived from the osteosarcoma cell line 143B.TK^-^. There were no significant differences of oxygen consumption, inhibition of cellular respiration by rotenone, and of complex IV enzymatic activity [47]. In accordance with the study of Carelli et al., we did not detect a significant difference in complex IV enzymatic activity. Also consistent with the results of Carelli et al., who found growth of haplogroup T cybrids in galactose medium to be “at the lower end of the range”, in our competitive mix experiments HEK T cybrids seemed to have a growth disadvantage in galactose medium compared to HEK H cybrids [47].

In another study, Caucasian haplogroups H and T cybrids generated from a human lung carcinoma cell line (A549.B2) also did not show functionally important bioenergetic differences [48]. However, ROS susceptibility was not analyzed.

In contrast to haplogroup cybrid comparisons performed by other laboratories [27]–[29], [47], [48], we decided not to use a cancer-derived cell line for cybrid production, as most cancer cells are known to change their energy-producing properties [49]. Therefore, we used the non-tumor cell line HEK293 for cybrid production. A further innovation of our approach was the use of platelets of patients with CAD carrying haplogroup T and healthy subjects carrying haplogroup H. Sequence analysis of the mtDNA of the cybrids revealed mainly mtDNA sequence variations between the haplogroup specific cybrids, which affect non-coding regions of the mtDNA. To our knowledge none of them has been described to be associated with a mitochondrial disease. However, we still cannot exclude that the variations affecting rRNA genes and the D-loop of the mtDNA are able to contribute to the differences observed between our cybrids.

Because there was no certain sequence variation consistently detectable in either HEK H or HEK T cybrids we hypothesize that the haplogroups themselves are responsible for the discriminative performance observed in the present study (Supplementary Figure S1 and S2). This is supported by the fact that no statistical significant differences were detected among cybrids of one haplogroup (cell survival 250 µM, 325 µM, 400 µM and 475 µM H_2_O_2_ in glucose medium as well as 200 µM and 250 µM in galactose medium; growth rates on day three and four in glucose medium).

Because in transmitochondrial cybrids the nuclear genetic background is excluded in all cases, effects of individual specific nuclear-mtDNA interactions are excluded. However, we cannot exclude that in mtDNA so far unknown epigenetic alterations can occur during disease progression, which are also still present in the cybrid lines.

Differences between haplogroup-specific cybrids have been observed, for instance in a recent study by Gómez-Durán et al., who compared haplogroup H with haplogroup Uk in cybrids derived from 143B.TK^-^ cells [27]. Gómez-Durán et al. found a higher mtDNA copy number in haplogroup H cybrids than in Uk cybrids. The higher mtDNA copy number in H cybrids was explained as resulting from higher ROS production by this haplogroup that would enhance mtDNA replication, as both haplogroup-specific cybrids decreased their mtDNA level after treatment with the antioxidant N-acetyl-cysteine, and the effect was larger for cybrids H. In the present study, mtDNA copy number of HEK T cybrids tended to be higher compared to HEK H cybrids.

In a study of Moreno-Loshuertos et al. [50], common mouse mitochondrial variants were compared in cybrid cells. All variants showed a similar level of respiration. The authors observed a compensatory mechanism of specific variants with a lower respiration capacity per molecule of mtDNA, which up-regulated mtDNA levels through ROS-signaling. These cells also possessed higher activity of the ROS defense enzyme catalase and slower growth in galactose containing medium compared to glucose containing medium.

In a similar way, HEK T cybrids of the present study might possess similar OXPHOS capacity (no differences in OXPHOS enzymatic activities), but lower respiration capacity per molecule of mtDNA, compared to HEK H cybrids. A compensatory mechanism, through ROS-induced up-regulation of mtDNA, adopted to overcome a slightly less efficient OXPHOS may be compatible with less growth in galactose medium. Moreover, a higher amount of antioxidative enzymes may be the reason for the HEK T cybrids to be more successful in buffering exogenous ROS exposure.

Mitochondrial haplogroup-specific differences were also analyzed in vivo. Martínez-Redondo et al. analyzed maximal oxygen uptake (VO_2max_), mitochondrial oxidative damage (mtOD), and mtDNA haplogroups in 81 healthy Spanish men. VO_2max_ was significantly lower in haplogroup J compared to haplogroup H individuals. When mtOD in skeletal muscle was assessed, oxidative damage was found to be significantly higher in haplogroup H individuals (p = 0.04) and there was a positive correlation between mtOD and VO_2max_ (p = 0.01) [51]. Hence, their study indicates a higher vulnerability of mitochondrial haplogroup H to ROS, as does ours.

Previous literature hints toward a lower uncoupling and higher ATP production through OXPHOS in mitochondrial haplogroup H. For instance, comparison of mitochondrial haplogroups H and T in sperm cells showed a significant reduction of complex IV activity in T sperm compared to H sperm (p = 0.0184), indicating a lower OXPHOS performance of haplogroup T [8]. Moreover, in peripheral leukocytes of patients with Huntington’s disease and haplogroup H, a significantly higher ATP concentration was found compared to non-H individuals with Huntington’s disease [52].

In conclusion, we were able to show that mitochondrial haplogroups H and T are functionally different in our model system. However, the functional differences between mitochondrial haplogroups and their consequences are far from being fully elucidated.

## Methods

### Cell Lines and Culture Conditions

The generation of cybrids used in the present study has been described previously [31]. Two clones per donor were used. Cells were maintained in Dulbecco’s modified Eagles’s Medium (DMEM) high glucose (4.5 g/l) (Sigma-Aldrich, D5648, St. Louis, Missouri, USA) supplemented with 10% fetal bovine serum (FBS) (PAA Laboratories, Pasching, Austria), 3.7 g/l sodium bicarbonate (Sigma-Aldrich, St. Louis, Missouri, USA), 1% Penicillin-Streptomycin-Amphotericin B mixture (Lonza, Basel, Switzerland), 2.5 mM sodium pyruvate (Sigma-Aldrich, St. Louis, Missouri, USA) and 1% MEM non-essential amino acid solution (Sigma-Aldrich, St. Louis, Missouri, USA). For the experiments, media without antibiotics were used. Galactose (glucose-free) medium was prepared using DMEM without glucose (Sigma-Aldrich, D5030, St. Louis, Missouri, USA) supplemented with 10% FBS (PAA Laboratories, Pasching, Austria), 3.7 g/l sodium bicarbonate (Sigma-Aldrich, St. Louis, Missouri, USA), 2.5 mM sodium pyruvate (Sigma-Aldrich, St. Louis, Missouri, USA), 1% MEM non-essential amino acid solution (Sigma-Aldrich, St. Louis, Missouri, USA), 0.9 g/l galactose (Sigma-Aldrich, St. Louis, Missouri, USA) and 0.584 g/l L-glutamine.

According to Gómez-Durán et al. [27], mtDNA copy number reaches stable levels in cybrids 20 passages after the cybridization process. Therefore, we used cybrid cells passaged between 15 and 25 times after the cybridization process.

### MtDNA Sequence Analysis

Sequence analysis of the mtDNA was performed from two overlapping PCR fragments (107 to 8561; 7401 to 276) generated by long range PCR [53]. PCR products were purified using ExoSAP-IT (USB, Cleveland, OH, USA), and sequencing was conducted using ABI PRISM® BigDye® Terminator v3.1 Cycle Sequencing Kit according to the manufacturer’s protocol (Applied Biosystems by Life Technologies, Carlsbad, California, USA) [54].

### Isolation of Mitochondria and Enzyme Measurements

Confluent cells were harvested, washed with phosphate-buffered saline (PBS), and mitochondria were isolated according to Bentlage et al. [55]. Enzyme activity measurements were performed as previously described [56], [57]. The protein content of isolated mitochondria was determined by BCA assay (Thermo Scientific, Rockford, Illinois, USA).

### Determination of mtDNA Copy Number

Cybrid cells were cultivated in glucose or galactose medium. DNA was isolated three times for each cybrid clone. The cell pellet was washed in PBS and subsequently resuspended in proteinase K-containing buffer [2 mg/ml proteinase K (Roche, Basel, Switzerland) in 1×Reaction Buffer B used for Hot Fire Polymerase (Solis Biodyne, Tartu, Estonia)]. After incubation for at least one hour at 60°C, proteinase K was inhibited by incubation at 95°C for 10 minutes.

MtDNA content was determined by qPCR using SYBR Green SuperMix for iQ (VWR International, Radnor, Pennsylvania, USA). Two mtDNA fragments and two nuclear DNA fragments were amplified using 0.2 µM of primers, 1×SYBR Green Supermix for iQ, 1 µl of DNA (diluted 1∶10 to 1∶40) in a total volume of 10 µl. Thermal cycling conditions were: 95°C for 1 minute; 40 cycles at 96°C for 15 seconds, 63°C for 40 seconds and 72°C for 10 seconds; and finally 95°C for 1 minute, 55°C for 1 minute and a 0.5°C increase per cycle (80×5 seconds) from 55°C to 95°C for the generation of a melting curve. Primer sequences are listed in Supplementary Table S2. MtDNA copy number was calculated with the following formula: 2^[mean Ct (nuclear fragments) – mean Ct (mitochondrial DNA fragments)]^.

### Determination of Growth Velocity

#### Growth curves

Over a period of six days the number of cybrid cells was measured by CyQUANT® NF Cell Proliferation Assay Kit (Invitrogen by Life technologies, Carlsbad, California, USA).

Cells were seeded at 1000 cells/ml in glucose medium and at 2500 cells/ml in galactose medium (200 µl per well) in a black 96-well plate. Cell number measurements were performed at 48, 72, 96, 120, 144 and 168 hours after seeding of the cells. We determined growth rates from day three to day seven, as there was no proliferation of cells observed before day three.

Measurements were carried out according to the manufacturer’s protocol using 50 µl dye solution and an incubation time of 60 minutes in the dark (37°C, 5% CO_2_). Fluorescence was measured (excitation: 490 nm, emission: 510–570 nm) on a GloMax®-Multi Microplate Multimode Reader (Promega, Madison, Wisconsin, USA).

The median of the fluorescence units of eight wells was calculated for each clone. Measurements of 72, 96, 120, 144, 168 hours were normalized to the value at 48 hours (determined as growth rate).

#### Competitive mix experiments

Each HEK H cybrid cell line was co-cultured in an initial 1∶1 ratio with each HEK T cybrid cell line either in glucose or galactose medium, resulting in a total of 36 co-culture combinations. After 10, 20 and 30 days of co-culture, the proportion of each genotype was determined by qPCR using TaqMan methods and probes specific for 7028C (haplogroup H) and 7028 T (non haplogroup H) [27].

The cells were seeded at 2500 cells/ml (glucose medium) or at 10,000 cells/ml (galactose medium) in 12-well plates (1 ml per well; 2 wells per co-culture). The medium was changed on days two, six, 12, 16, 22, 26 or additionally if necessary. Before reaching confluency, on days 10 and 20, co-cultures were trypsinized and sub-cultured. DNA was isolated on days 10, 20 and 30.

The genotype proportion in the mix experiments was determined by qPCR, using TaqMan® Gene Expression Master Mix and TaqMan reagents (Applied Biosystems by Life Technologies, Carlsbad, California, USA). Primer and probe sequences are listed in Supplementary Table S2 [27].

The PCR mixture contained 0.9 µM primers, 0.05 µM 7028C probe (VIC-labelled), 0.1 µM 7028 T probe (FAM-labelled), 1×TaqMan® Gene Expression Master Mix and 1 µl of DNA (diluted 1∶10 to 1∶100) in a total volume of 15 µl. Thermal cycling conditions were: 95°C for 8.5 minutes and 40 cycles at 95°C for 15 seconds and 65°C for 1 minute.

The proportion of a specific haplogroup in the mixture was calculated with the following formula: ΔC_t_ = C_t_ (VIC)−C_t_ (FAM). Positive ΔC_t_ values stand for a higher ratio of haplogroup T in the sample and negative ΔC_t_ values reveal a higher fraction of haplogroup H. ΔΔC_t_ values represent ΔC_t_ values at days 10, 20 or 30 minus the initial ΔC_t_ values (day zero; only one mixture).

In cases where only the signal of one probe was detected, as this haplogroup has probably displaced the other, the C_t_ value of the second probe was set to 31, in order to be able to calculate a shift. This value was determined as the highest detectable C_t_ value was 31.

As two wells were seeded per co-culture, the mean of both wells was calculated and used as the C_t_ value of the respective mixture in the statistical analysis.

### Determination of Susceptibility to ROS

Cells were seeded in 96-well plates at 10^5^ cells/ml in glucose medium (200 µl per well). Twenty-four hours after seeding, the medium was changed. For ROS susceptibility experiments, medium without serum and without sodium pyruvate was used in order to avoid potential antioxidative components in the medium [58], [59].

Twenty-four hours after the change of medium, the cells were treated with different concentrations of H_2_O_2_. For each concentration of H_2_O_2_, eight wells were treated per clone. Cell survival was analyzed after 24 hours using resazurin (resazurin sodium salt, Sigma-Aldrich, St. Louis, Missouri, USA). Resazurin is reduced in living cells to the pink fluorescent dye resorufin [60]. Forty microliters of resazurin (2.5 mM in 1×PBS) were added to each well, the plates were incubated for 2 hours in the dark (37°C, 5% CO_2_) and fluorescence was measured (excitation: 525 nm, emission: 580–640 nm) on a GloMax®-Multi Microplate Multimode Reader (Promega, Madison, Wisconsin, USA).

The median of the fluorescence units of eight blank wells (only medium with dye) was subtracted from the median of the fluorescence units of the eight wells per clone. Cell survival at a certain concentration of H_2_O_2_ was calculated as the percentage of the fluorescence units of the treated cells in relation to non-treated cells.

### Statistical Analysis

Normal distribution was checked by the Kolmogorov-Smirnov test. Differences between mean enzymatic activities, mtDNA copy number, growth rates and cell survival upon challenge with H_2_O_2_ were statistically evaluated using an independent samples t-test (for normally distributed variables) or a non-parametric Mann-Whitney U-test (for not normally distributes variables). In the competitive mix experiments, variables were analyzed using a dependent samples t-test (for normally distributed variables) or a Wilcoxon signed-rank test (for not normally distributed variables). A p-value<0.05 was considered statistically significant. All analyses were performed using PASW Statistics 18.0 (SPSS GmbH).

## Supporting Information

Figure S1 **Growth rates of single cybrids in glucose medium on day three and day four.**
(PDF)

Figure S2 **Cell survival of single cybrids after treatment with hydrogen peroxide.**
(PDF)

Table S1 **MtDNA polymorphisms of cybrids, not used for classification of mitochondrial sub-haplogroups and not present in all cybrids.**
(PDF)

Table S2 **Primers used in quantitative PCR experiments.**
(PDF)
