# Supplementary material for: Functional Differences between Mitochondrial Haplogroup T and Haplogroup H in HEK293 Cybrid Cells
Source: PLoS One. 2012 Dec 26;7(12):e52367. doi: 10.1371/journal.pone.0052367 (PMC3530588; doi:10.1371/journal.pone.0052367)
Supplement: Figure S1 — Growth rates of single cybrids in glucose medium on day three and day four. (PDF) [file pone.0052367.s001.pdf]

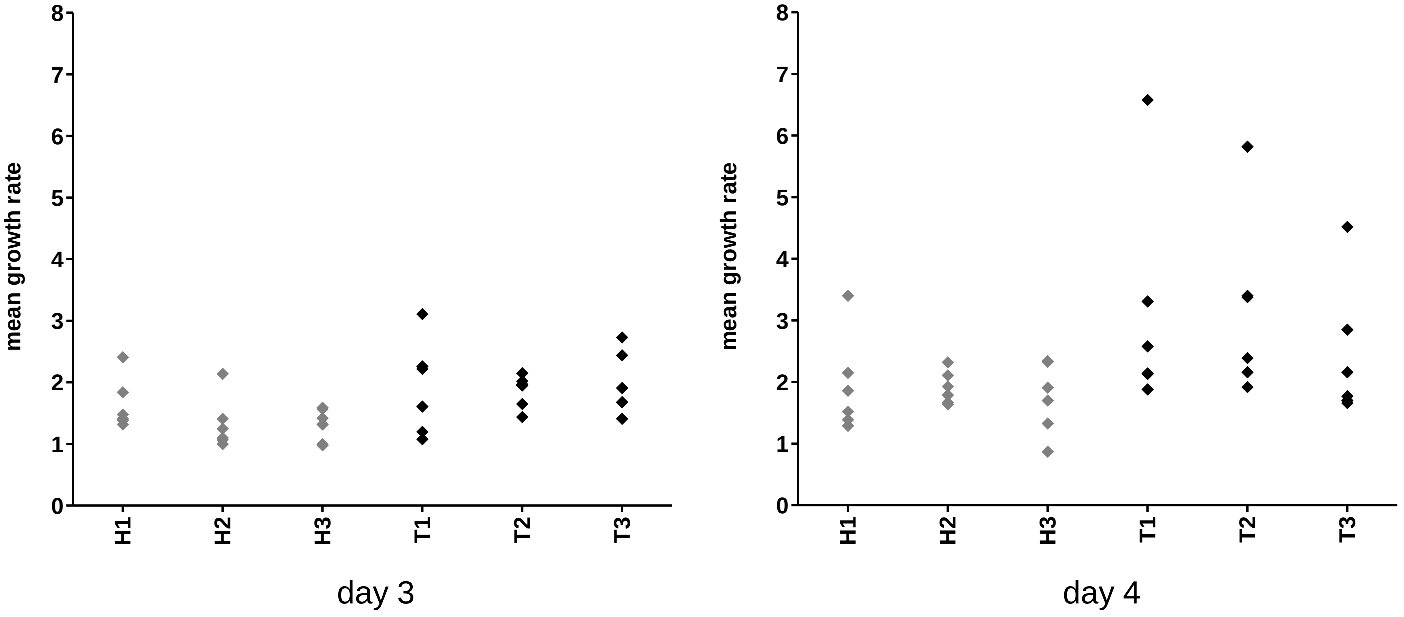

Supplementary Figure S1. Growth rates of single cybrids in glucose medium on day three and day four. The number of cells on the days given were normalized to the number of cells on day two and determined as growth rate. Haplogroup H cybrids are shown as gray circles and haplogroup T cybrids are shown as black squares.
