# Supplementary material for: Functional Differences between Mitochondrial Haplogroup T and Haplogroup H in HEK293 Cybrid Cells
Source: PLoS One. 2012 Dec 26;7(12):e52367. doi: 10.1371/journal.pone.0052367 (PMC3530588; doi:10.1371/journal.pone.0052367)
Supplement: Figure S2 — Cell survival of single cybrids after treatment with hydrogen peroxide. (PDF) [file pone.0052367.s002.pdf]

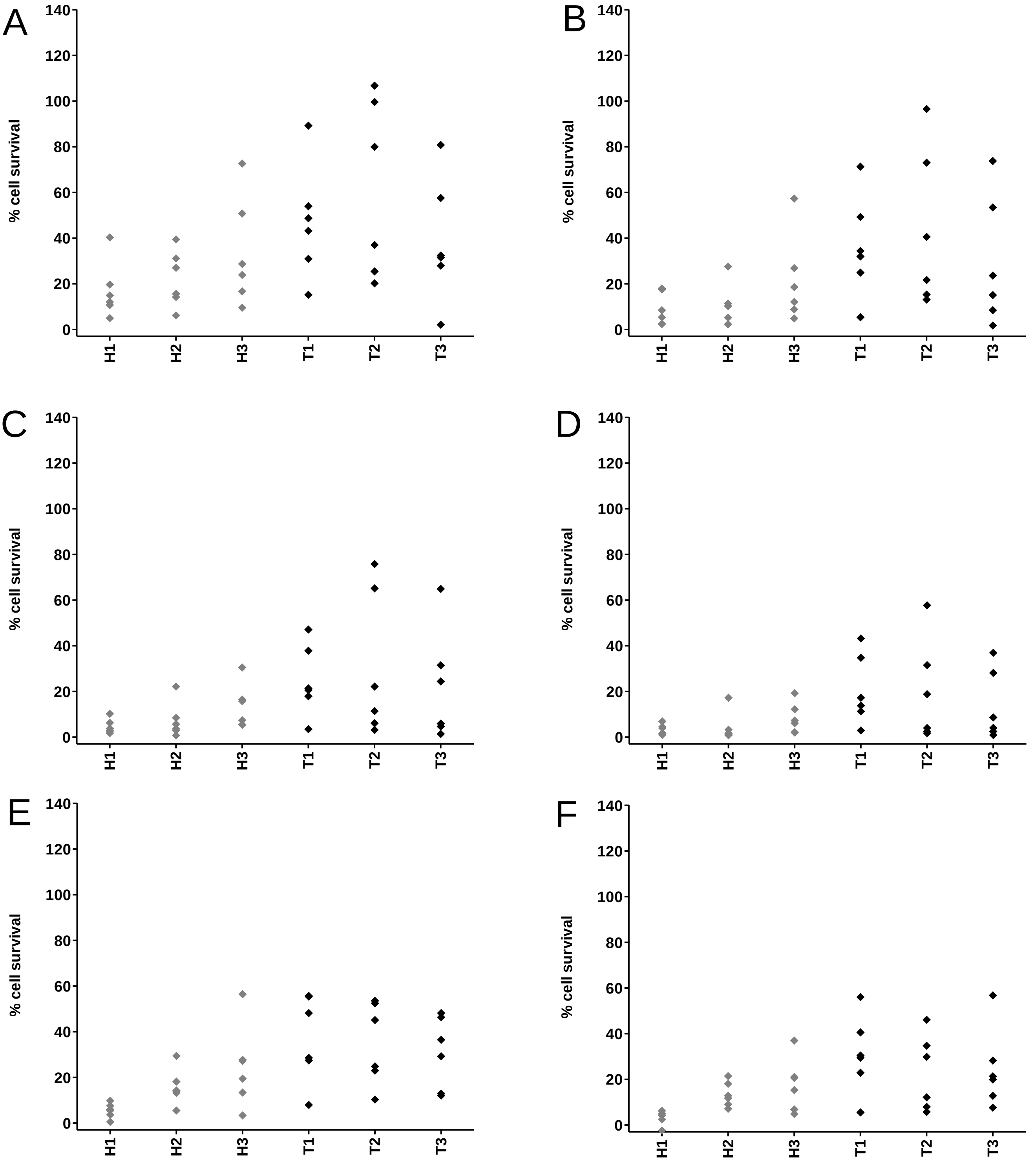

Supplementary Fig S2. Cell survival of single cybrids after treatment with hydrogen peroxide.
Cell survivial of haplogroup H (gray rectangles) and haplogroup T (black rectangels) cybrids was measured 24 hours after H_2O_2 treatment and calculated as percentage to cells without treatment (% cell survival). (A) Cell survival in glucose medium without serum and without sodium pyruvate and treated with 250 µM H_2O_2. (B) Cell survival in glucose medium without serum and without sodium pyruvate and treated with 325 µM H_2O_2. (C) Cell survival in glucose medium without serum and without sodium pyruvate and treated with 400 µM H_2O_2. (D) Cell survival in glucose medium without serum and without sodium pyruvate and treated with 475 µM H_2O_2. (E) Cell survival in galactose medium without serum and without sodium pyruvate and treated with 200 µM H_2O_2. (F) Cell survival in galactose medium without serum and without sodium pyruvate and treated with 250 µM H_2O_2.
