# Supplementary material for: Functional Differences between Mitochondrial Haplogroup T and Haplogroup H in HEK293 Cybrid Cells
Source: PLoS One. 2012 Dec 26;7(12):e52367. doi: 10.1371/journal.pone.0052367 (PMC3530588; doi:10.1371/journal.pone.0052367)
Supplement: Table S1 — MtDNA polymorphisms of cybrids, not used for classification of mitochondrial sub-haplogroups and not present in all cybrids. (PDF) [file pone.0052367.s003.pdf]

Supplementary Table S1. MtDNA polymorphisms of cybrids, not used for classification of mitochondrial sub-haplogroups and not present in all cybrids.

Cybrid cell line	Polymorphism	MtDNA location	Amino acid change
**H1**	m.5899-m.5900insC	non-coding nucleotides	noncoding
**H2**	m.6996A>C[b] (46% substitution)[c]	MT-CO1	p.Ile365Leu[a]
	m.15246G>A[b] (26% substitution)[c]	MT-CYB	p.Gly167Asp[a]
	m.16519T>C	D-Loop	noncoding
**H3**	m.152T>C	D-Loop	noncoding
	m.2170G>A[b] (48% substitution)[c]	MT-RNR2	coding[d]
	m.2412A>G[b]	MT-RNR2	coding[d]
**T1**	m.1760G>A[b]	MT-RNR2	coding[d]
	m.12696T>C	MT-ND5	syn
	m.16519T>C	D-Loop	noncoding
**T2**	m.573-m.574insC	D-Loop	noncoding
	m.1598G>A	MT-RNR1	coding[d]
	m.14324T>C	MT-ND6	p.Asn117Ser
	m.16519T>C	D-Loop	noncoding
**T3**	m.151C>T	D-Loop	noncoding
	m.152T>C	D-Loop	noncoding
	m.16184C>T	D-Loop	noncoding
	m.16280A>G	D-Loop	noncoding
	m.16344C>T	D-Loop	noncoding
	m.16519T>C	D-Loop	noncoding

Polymorphisms are compared to the Revised Cambridge Reference Sequence.
[a]in case of base change.
[b]Polymorphism not listed in MITOMAP (www.mitomap.org) and/or the Human Mitochondrial Genome Database (www.genpat.uu.se/mtDB/).
[c]heteroplasmic position.
[d]coding for RNA.
MT-CO1=Cytochrome c oxidase subunit 1.
MT-CYB=Cytochrome b.
MT-RNR1=12S ribosomal RNA.
MT-RNR2=16S ribosomal RNA.
MT-ND5=NADH dehydrogenase subunit 5.
MT-ND6=NADH dehydrogenase subunit 6.
syn=synonymous mutation.
