# Supplementary material for: Functional Differences between Mitochondrial Haplogroup T and Haplogroup H in HEK293 Cybrid Cells
Source: PLoS One. 2012 Dec 26;7(12):e52367. doi: 10.1371/journal.pone.0052367 (PMC3530588; doi:10.1371/journal.pone.0052367)
Supplement: Table S2 — Primers used in quantitative PCR experiments. (PDF) [file pone.0052367.s004.pdf]

Supplementary Table S2. Primers used in quantitative PCR experiments.

forward	*reverse*
Primers for quantification of mtDNA copy number	
mtDNA1-D AGCCGCCGCCTGATACTG	mtDNA1-R GGGGATATAGGGTCGAAGCCG
mtDNA2-D CGGAGGAGGAGACCCCATTC	mtDNA2-R TGGTAGCGGAGGTGAAATATGC
nDNA1-D CGAGTAGTCAGTGTCCAAGAAATGG	nDNA1-R AATGGCTTGCATCAGCTTATGTTC
nDNA2-D CCAGCATTTCCTCTTCCCTGTT	nDNA2-R CTCCTCAAGAGATCTCCCACCC
Primers for competitive mix experiment	
mtDNA-D GCAAACTCATCACTAGACATCGTACT	mtDNA-R CCTATGATGGCAAATACAGCTCCTAT
Probes for competitive mix experiment	
7028C TAGTGGAAGTGGGCTACAA	7028T TAGTGGAAGTGAGCTACAA

Primers are listed in 5‘→3‘ direction
